# Racial differences in male fertility parameters in 2,996 men examined for infertility in a single center

**DOI:** 10.1080/20905998.2025.2470045

**Published:** 2025-02-23

**Authors:** Ralf Henkel, Haitham Elbardisi, Ahmad Majzoub, Mohamed Arafa

**Affiliations:** a LogixX Fertility Section, LogixX Pharma, Theale, Berkshire, UK; b Department of Medical Biosciences, University of the Western Cape, Bellville, South Africa; c Department of Metabolism, Digestion and Reproduction, Imperial College London, London, UK; d Urology Department, Hamad Medical Corporation, Doha, Qatar; e Urology Department, Weill Cornell Medicine-Qatar, Doha, Qatar; f Andrology Department, Cairo University, Cairo, Egypt

**Keywords:** Racial differences, semen parameters, sperm DNA fragmentation, oxidative stress, hormones

## Abstract

**Objectives:**

Several studies have demonstrated racial variations in various diagnostic clinical parameters in different fields of medicine including andrology. Yet, clinical andrological diagnostic is following the lower reference values recommended by the World Health Organization irrespective of the racial descent of men. Therefore, this study aimed to investigate racial differences in hormonal and semen parameters including sperm DNA fragmentation (SDF) and seminal oxidative stress in a large group of patients from Sub-Saharan, Caucasian, Central/South Asian, Middle Eastern, North African, and Southeast Asian descent.

**Methods:**

In a total of 2,996 infertile men, testis volume, sperm concentration, total sperm count, total and progressive motility, normal morphology, sperm vitality, SDF, oxidation-reduction potential (ORP), and standard hormones were determined and compared.

**Results:**

Significant racial differences for various parameters including the incidence of normal ranges values in the racial groups were found. The highest mean sperm concentration had men of Central/South Asian descent (median: 38.0 × 10^6^/mL) while Southeast Asian men had the lowest (median: 22.0 × 10^6^/mL; *p* < 0.0001). The highest total sperm motility (median: 55.0%) was observed in Caucasian, Central/South Asian, and Southeast Asian men, while Sub-Saharan African men had the lowest (median: 45.0%; *p* < 0.0001). For SDF, Caucasian men had the lowest sperm DNA fragmentation (median: 16.0%) and ORP values (median: 1.3 mV/10^6^ sperm/mL) as compared to Central/South Asian men (median: 28.0%; *p* = 0.0263) and Southeast Asian men (median: 2.4 mV/10^6^ sperm/mL; *p* = 0.0045), respectively.

**Conclusions:**

Our results show not only significant racial differences for many of the parameters investigated but also for the incidence of normal values. Therefore, it might be prudent to revisit the concept of globally standardized reference values for all men. Yet, as a limitation, the small number (53) of Caucasian men needs to be mentioned. Larger studies that include functional sperm parameters need to be conducted.

## Introduction

Infertility is a globally underestimated and under-recognized public health condition reportedly affecting approximately 15% of couples at reproductive age at some point in their lifetime [1]. This amounts to an estimate of 186 million infertile couples with an estimate of 93 million infertile men [2]. Male fertility evaluation according to World Health Organization (WHO) criteria includes semen analysis, hormone analysis, and physical examination [3,4]. Initially, WHO semen analysis protocols relied mainly on data from Northern Europe and North America, leading to over-representation of Caucasian men [5]. The 2010 5th Edition of the WHO manual included samples from over 1953 men across 8 countries and 3 continents [6]. The latest Edition expanded this to over 3500 men with greater diversity [7].

All these diagnostic procedures are standardized with normal values/lower reference values, which apply globally, regardless of ethnicity. However, several studies have demonstrated racial variations in semen parameters such as sperm concentration, motility, and normal sperm morphology with men of African or Asian descent having lower semen quality than white men [8–12]. Elbardisi et al. in a study including 13,892 men attending the Male Infertility Unit at Hamad Medical Corporation, in Doha, Qatar, report that men from the Middle East and North Africa (MENA) region have a lower semen quality than men from other regions [13]. On the other hand, other studies showed no racial differences [14]. Most studies that investigated racial differences have compared only semen parameters of men of two or three different racial backgrounds or compared different patient groups from different centers.

Therefore, in order to obtain a more comprehensive overview, it was the aim of this study to investigate racial differences in diagnostic parameters for male infertility not only for semen parameters such as sperm concentration, motility, or morphology in two or three racial groups, but a wider range of different diagnostic parameters including testicular volume, seminal oxidative stress, sperm DNA fragmentation, and hormonal parameters. Moreover, this study aimed at investigating these parameters in men with different racial descent in a single-center setup so that possible inter-laboratory variations as well as environmental and geographical influences like temperature and location on northern or southern hemisphere, respectively, could largely be avoided. If there are indeed racial differences in semen and hormonal parameters, it would make sense to adjust them accordingly in order to diagnose the patient properly in a more personalized approach.

## Materials and methods

### Patients

In this retrospective study, a total of 2,998 men aged between 15 and 73 years attending the Department of Urology at Hamad Medical Corporation, Doha, Qatar, between 1 January 2016 and 1 January 2018 for male infertility were evaluated with no specific categories for primary and secondary infertility. The patients were then categorized according to the region of their origin, Sub-Saharan African, North African, Caucasian, Middle East, Central/South Asia, and Southeast Asia. Considering that only 2 patients were from East Asia (Chinese), this group was not considered further, thus a total of 2,996 patient were included in this study.

Apart from the standard semen parameters (sperm concentration, total and progressive motility, normal sperm morphology, and vitality), testicular volume, and serum levels of estradiol, follicle stimulating hormone (FSH), luteinizing hormone (LH), prolactin, and testosterone were determined. In addition, sperm DNA fragmentation (SDF), and oxidation-reduction potential (ORP) were determined.

### Semen analysis

Semen samples were collected in sterile plastic containers after 3–5 days of sexual abstinence. Samples were liquefied at 37°C for 30 min and standard semen analysis was performed according to 2010 World Health Organization guidelines.^5^

### Determination of testicular volume

Determination of the testicular volume was performed by a consultant radiologist using scrotal ultrasonography using a 7.5 MHz probe. The length (longitudinal diameter), width (transverse diameter), and height (anterior posterior diameter) of the testes were measured. Subsequently, the testicular volume was calculated using the formula for an ellipsoid formula: length (L) × width (W) × height (H) × 0.52.

### Determination of sperm DNA fragmentation

Sperm DNA fragmentation (SDF) was measured using the Halosperm G2 Test kit (Halotech DNA SL, Madrid, Spain). With this test kit, SDF is determined by means of sperm chromatin dispersion, a process which involves the denaturation and controlled lysis of the sample in an appropriate medium. Sperm with intact DNA produces a dispersion halo because of the chromatin released from nuclear proteins. The result can then be analyzed using fluorescence or bright field microscopy. Sperm with fragmented DNA will not produce this halo [15].

### Determination of seminal oxidative stress

Seminal oxidative stress was measured by means of the MiOXSYS system (Aytu BioScience, Englewood, CO), a galvanostat-based technology that measures the oxidation-reduction potential (ORP). An aliquot of 30 µL of liquefied semen was applied to the disposable sensor inserted in the device. The ORP value is displayed on the screen after about 4 min in millivolts (mV). Afterwards, the ORP readings were normalized according to the seminal sperm concentration and expressed as mV/10^6^ sperm/mL [16].

### Hormone analysis

Blood samples for hormonal assay were collected from the patients on the day of visit between 7:00 and 9:00 am. The analysis was done in the endocrinology laboratory of Hamad Medical Center using the immunoassay chemiluminescence method, Architect i1000SR^Ⓡ^ (Abbott Systems, Illinois, USA). The hormonal profile included follicular stimulating hormone (FSH), luteinizing hormone (LH), prolactin, total testosterone, and estradiol.

### Statistical analysis

Statistical analysis was performed using MedCalc statistical software (v 22.023; MedCalc Software Ltd., Ostend, Belgium). After checking for normal distribution of the data by means of the Shapiro–Wilk test, non-parametric tests (Mann–Whitney) and odds ratios were applied and data presented accordingly. For further statistical analysis, patients were categorized according to WHO criteria in normozoospermia, oligozoospermia, asthenozoospermia, and teratozoospermia [5]. Categorization for FSH level (low: <1.0 IU/L; normal: 1.0 to 18.0 IU/L; high: >18.0 IU/L), LH level (low: <1.8 IU/L; normal: 1.8 to 8.6 IU/L; high: >8.6/L) and normal total testis volume (small: <18 mL; normal: ≥18 mL) was done as described previously by Arafa et al. [17]. The reference values for the other hormones were as follows: prolactin (low: <85.0 mIU/L, normal 85.0 to 323.0 mIU/L, high: >323.0 mIU/L), testosterone (low: <10.4 nmol/L, normal: 10.4 to 37.44 nmol/L, high: >37.44 nmol/L), and estradiol (low: <41.4 pmol/L, normal: 41.4 to 159.0 pmol/L, high: >159.0 pmol/L). In addition, for ORP, patients were categorized into normal (<1.34 mV/10^6^ sperm/mL) and high (≥1.34 mV/10^6^ sperm/mL) according to Agarwal et al. [18]. An SDF level of ≥30% was accepted as high [19].

Considering that variables such as sperm concentration, testosterone, and ORP are also influenced by age, logistic regression analyses including Hosmer and Lemeshow tests for the fit of the logistic regression model were conducted to investigate the contribution of the racial background.

Statistical significance was defined as *p* < 0.05.

## Results

Of the total of 2,996 patients included in the study, 1,279 (42.7%) men came from the Middle East, 692 (23.1%) from Northern Africa, 611 (20.4%) from Central/South Asia, 125 (4.2%) from Southeast Asia, while 176 (5.9%) were of Sub-Saharan African and 53 (1.8%) of Caucasian descent. For the testicular volume, the highest values with a median of 20.3 mL were found in Central/South Asian men, whereas Southeast Asian men had the lowest (median: 9.6 mL). This difference was significant (*p* = 0.0233). Summary results for all analyzed parameters are depicted in Table 1.

**Table 1.** Summary statistics for various parameters of men from different regions (*n* = 3,913).

	Sub-Saharan African			Caucasian			Central/South Asian			Middle East			North African			Southeast Asian		
	n	Median	IQR	n	Median	IQR	n	Median	IQR	n	Median	IQR	n	Median	IQR	n	Median	IQR
Age (yrs)	176	38.0	33.0–46.0	53	39.0	33.7–46.3	611	35.0	31.0–39.0	1279	35.0	30.0–40.0	692	34.0	30.0–38.0	125	36.0	33.8–39.0
Total testis volume (mL)	33	21.1	14.8	7	18.2	12.7–29.9	119	20.2	13.4–28.7	192	21.2	15.1–28.3	145	22.6	16.2–34.8	26	14.4	9.4–21.5
Sperm concentration (10^6^/mL)	176	25.5	9.5–40.0	53	26.0	11.8–49.5	611	38.0	18.0–58.0	1280	23.0	10.0–42.0	694	30.0	12.0–49.0	125	22.0	10.5–37.0
Total sperm count (10^6^)	176	60.0	21.0–120.0	53	65.0	33.2–107.3	610	90.0	40.5–160.0	1280	55.7	22.5–111.0	694	80.0	30.0–137.5	125	44.0	17.8–82.5
Total motile sperm count (10^6^)	176	25.6	5.6–64.9	53	32.0	13.9–65.7	610	45.0	17.6–90.0	1279	27.0	7.9–60.0	694	39.5	10.8–74.4	125	27.0	7.8–46.2
Total motility (%)	176	45.0	30.0–60.0	53	55.0	49.0–62.0	611	55.0	40.0–62.0	1278	50.0	35.0–60.0	694	50.0	40.0–60.0	125	55.0	40.0–65.0
Progressive motility (%)	176	5.0	0.0–15.0	53	15.0	5.0–25.0	611	10.0	0.0–20.0	1278	10.0	0.0–20.0	694	10.0	0.0–20.0	125	10.0	0.0–20.0
Normal sperm morphology (%)	176	3.0	1.0–5.0	53	5.0	3.0–6.2	611	4.0	2.0–6.8	1280	3.0	1.0–5.0	694	3.0	1.0–5.0	125	3.0	2.0–5.0
Sperm vitality (%)	57	47.0	27.3–59.0	5	50.0	28.8–71.3	100	45.0	30.0–60.0	303	50.0	35.0–60.0	160	47.5	30.0–60.0	20	51.5	30.0–62.5
Estradiol (pmol/L)	53	105.0	75.8–136.3	21	90.0	64.0–111.3	167	100.0	75.2–129.0	387	97.0	71.3–127.0	229	100.0	75.8–126.0	38	103.0	82.0–147.0
FSH (IU/L)	61	3.8	2.4–6.8	22	3.5	2.0–6.0	187	3.9	2.3–6.1	424	3.1	2.1–5.6	264	3.0	2.0–5.0	40	6.0	3.2–10.2
LH (IU/L)	61	4.0	3.0–6.0	22	3.6	2.2–5.1	187	3.6	2.5–5.0	425	3.8	2.8–5.4	262	3.7	2.7–4.9	41	3.3	2.9–5.1
Prolactin (mIU/L)	61	197.0	155.7–264.5	21	171.6	140.4–220.2	186	187.8	144.0–264.8	414	203.9	145.0–287.1	254	188.5	141.3–257.4	42	204.7	143.2–294.5
Testosterone (nmol/L)	61	14.5	12.2–19.4	23	17.4	12.5–24.5	189	15.4	11.0–19.0	425	17.0	13.1–23.1	259	17.1	13.1–22.7	41	16.2	12.6–20.3
Sperm DNA fragmentation (%)	42	25.0	20.0–38.0	13	16.0	13.0–25.5	157	28.0	17.0–43.0	331	23.0	15.0–33.0	176	25.0	15.0–42.0	29	18.0	11.5–28.5
ORP (mV/10^6^ sperm/mL)	176	2.2	1.1–6.6	53	1.3	0.7–3.7	611	1.5	0.9–3.0	1280	2.1	1.1–5.0	694	1.9	1.0–4.4	125	2.4	1.2–5.0

Overall, 247 (8.2%) patients presented with normozoospermia whereas oligo-, astheno-, terato-, oligo-astheno-, oligo-astheno-terato-, oligo-terato-, and astheno-teratozoospermia were diagnosed in 851 (28.4%), 2670 (89.1%), 1535 (51.2%), 106 (3.5%), 739 (24.7%), 15 (0.5%), and 805 (26.9%) patients, respectively (Table 2).

**Table 2.** Proportions of patients with described clinical conditions among different racial groups of patients.

	Sub-Saharan African % (positive/racial group)	Caucasian % (positive/racial group)	Central/South Asian % (positive/racial group)	Middle East % (positive/racial group)	North African % (positive/racial group)	Southeast Asian % (positive/racial group)
Normal testicular volume	57.6 (19/33)	85.7 (6/7)	59.7 (71/119)	63.0 (121/192)	66.2 (96/145)	38.5 (10/26)
Normozoospermia	7.4 (13/176)	17.0 (9/53)	10.3 (63/611)	7.8 (100/1280)	7.6 (53/694)	7.2 (9/125)
Oligozoospermia	30.1 (53/176)	26.4 (14/53)	19.3 (118/611)	32.7 (418/1280)	29.6 (205/694)	34.4 (43/125)
Asthenozoospermia	92.6 (163/176)	81.1 (43/53)	89.2 (545/611)	91.6 (1172/1279)	91.1 (631/694)	92.8 (116/125)
Teratozoospermia	60.8 (107/176)	35.8 (19/53)	43.5 (266/611)	54.3 (695/1280)	54.7 (379/694)	55.2 (69/125)
Oligo-asthenozoospermia	0.7 (1/176)	7.5 (4/53)	2.6 (16/611)	4.3 (55/1280)	3.0 (21/694)	4.0 (5/125)
Oligo-astheno-teratozoospermia	29.0 (51/176)	17.0 (9/53)	16.9 (103/611)	27.6 (353/1280)	25.5 (177/694)	30.4 (38/125)
Oligo-teratozoospermia	0.0 (0/176)	0.0 (0/53)	0.0 (0/611)	0.3 (4/1280)	0.3 (2/694)	0.0 (0/125)
Astheno-teratozoospermia	32.4 (57/176)	18.9 (10/53)	27.3 (167/611)	26.7 (342/1280)	28.5 (198/694)	25.6 (32/125)
Sperm DNA fragmentation High	35.7 (15/42)	15.4 (2/13)	44.6 (70/157)	30.5 (101/331)	38.6 (68/176)	24.1 (7/29)
ORP High	70.5 (124/176)	45.3 (24/53)	56.5 (345/611)	67.0 (858/1280)	62.8 (436/694)	70.4 (88/125)
Estradiol Low Normal High	0.0 (0/53) 84.9 (45/53) 15.1 (8/53)	0.0 (0/21) 95.2 (20/21) 4.8 (1/21)	2.4 (4/167) 86.8 (145/167) 10.8 (18/167)	4.4 (17/387) 83.5 (323/387) 12.1 (47/387)	3.0 (7/229) 86.5 (198/229) 10.5 (24/229)	2.6 (1/38) 84.2 (32/38) 13.2 (5/38)
Follicle stimulating hormone Low Normal High	4.9 (3/61) 85.3 (52/61) 9.8 (6/61)	18.2 (4/22) 72.7 (16/22) 9.1 (2/22)	9.1 (17/187) 86.6 (162/187) 4.3 (8/187)	11.6 (49/424) 84.4 (358/424) 4.0 (17/424)	9.8 (26/264) 85.6 (226/264) 4.6 (12/264)	5.0 (2/40) 80.0 (32/40) 15.0 (6/40)
Luteinizing hormone Low Normal High	1.7 (1/60) 91.7 (55/60) 6.6 (4/60)	18.2 (4/22) 81.8 (18/22) 0.0 (0/22)	7.0 (13/187) 87.7 (164/187) 5.3 (10/187)	7.5 (32/424) 86.3 (366/424) 6.2 (26/424)	5.3 (14/262) 87.8 (230/262) 6.9 (18/262)	4.9 (2/41) 90.2 (37/41) 4.9 (2/41)
Prolactin Low Normal High	4.9 (3/61) 78.7 (48/61) 16.4 (10/61)	14.3 (3/21) 76.2 (16/21) 9.5 (2/21)	4.3 (8/186) 79.6 (148/186) 16.1 (30/186)	3.9 (16/414) 76.8 (318/414) 19.3 (80/414)	3.1 (8/254) 84.3 (214/254) 12.6 (32/254)	0.0 (0/42) 78.6 (33/42) 21.4 (9/42)
Testosterone Low Normal High	14.8 (9/61) 83.6 (51/61) 1.6 (1/61)	21.7 (5/23) 73.9 (17/23) 4.3 (1/23)	20.1 (38/189) 79.4 (150/189) 0.5 (1/189)	11.1 (47/425) 84.7 (360/425) 4.2 (18/425)	11.6 (30/259) 85.7 (222/259) 2.7 (7/259)	14.6 (6/41) 85.4 (35/41) 0.0 (0/41)

**Figure 1.**
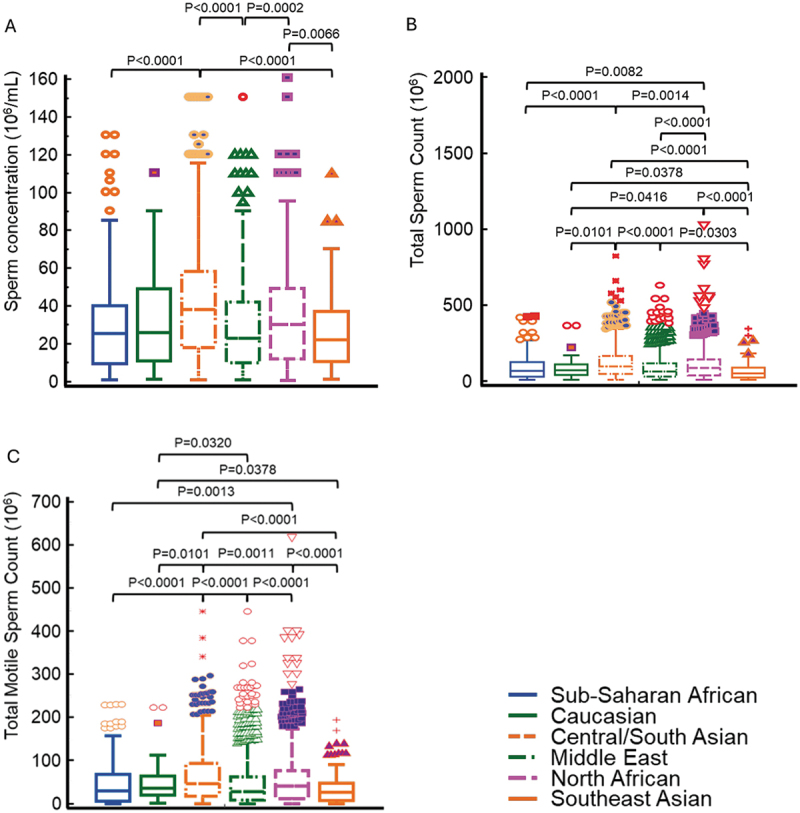
Box-and-whisker plots of sperm concentration (a), total sperm count (b), and total motile sperm count (c) of men from different regions. Significant differences between men coming from different regions are obvious.

**Figure 2.**
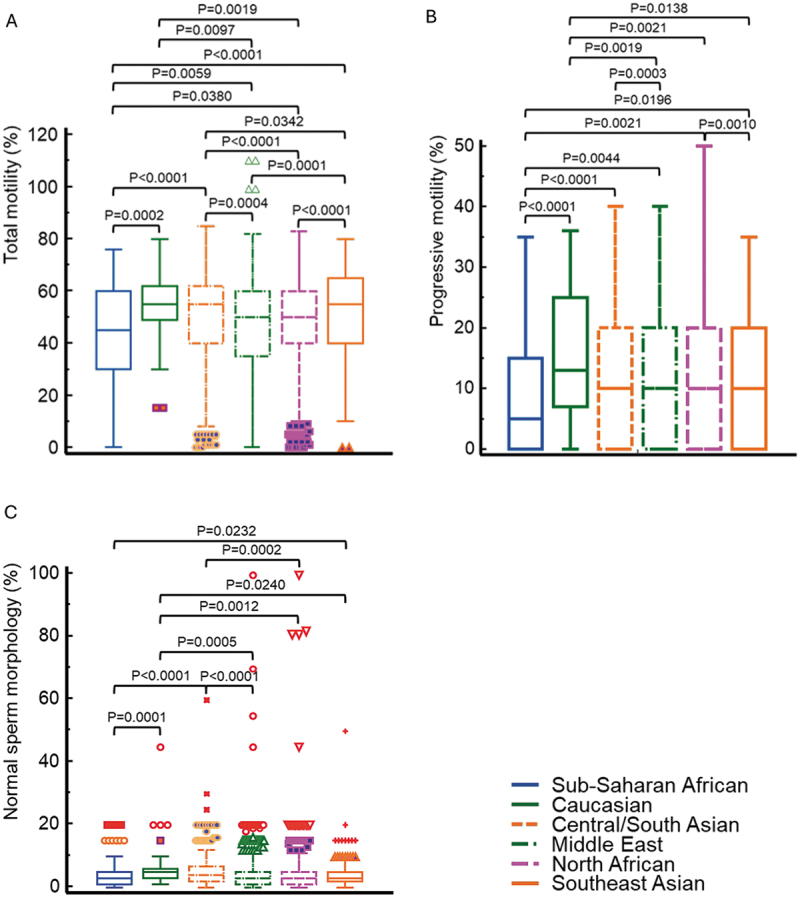
Box-and-whisker plots of total motility (a), progressive motility (b), and normal sperm morphology (c) of men from different regions. Significant differences between men coming from different regions are obvious.

Results show significant differences for sperm concentration between different racial groups with Central/South Asian men exhibiting the highest values while Southeast Asians had the lowest (*p* < 0.0001) (Figure 1a). For the total sperm count (Figure 1b) and total motile sperm count (Figure 1c) highest values were observed for Central/South Asian men, whereas the lowest values had Southeast Asian men and Sub-Saharan men, respectively (Table 1). Similarly, significant differences between men of different racial descent were observed for total and progressive motility, and normal sperm morphology (Table 1; Figure 2a–c).

**Figure 3.**
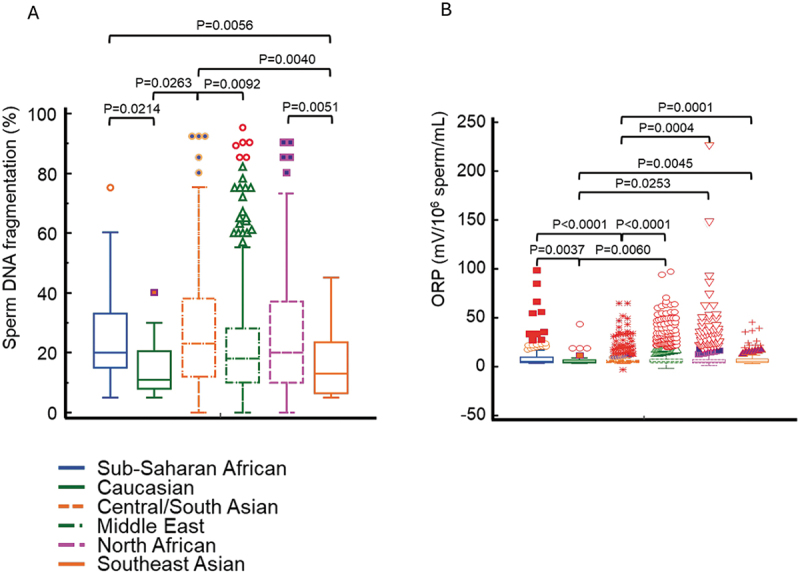
Box-and-whisker plots of sperm DNA fragmentation (a) as determined with the HaloSperm kit and seminal oxidative stress as determined as oxidation-reduction potential (ORP) (b) by means of the MiOXSYS system of men from different regions. Significant differences between men coming from different regions are obvious.

Concerning sperm functional aspects, significant differences were also observed for sperm DNA fragmentation (Figure 3a) and seminal oxidative stress as determined by means of ORP (Figure 3b). The proportions of patients of the respective racial groups with normo-, oligo-, astheno-, and teratozoospermia, as well patients with normal testicular volume, low, normal, and high hormonal concentrations, high sperm DNA fragmentation, and seminal oxidative stress levels as measured by means of ORP are depicted in Table 2.

Men from Central/South Asia showed the highest probability of having elevated levels of sperm DNA fragmentation, the lowest was observed in Caucasian men with the odds being 2.9 times higher, yet not significant (*p* = 0.1687). The highest probability of having high seminal oxidative stress levels had Sub-Saharan Africans, while the lowest probability for elevated ORP values was observed in Caucasians with significantly higher odds as compared to Caucasians (OR: 1.56; *p* = 0.1048), Southeast Asians (OR: 1.55; *p* = 0.1185), and Middle Eastern men (OR: 1.48; *p* = 0.1166).

**Figure 4.**
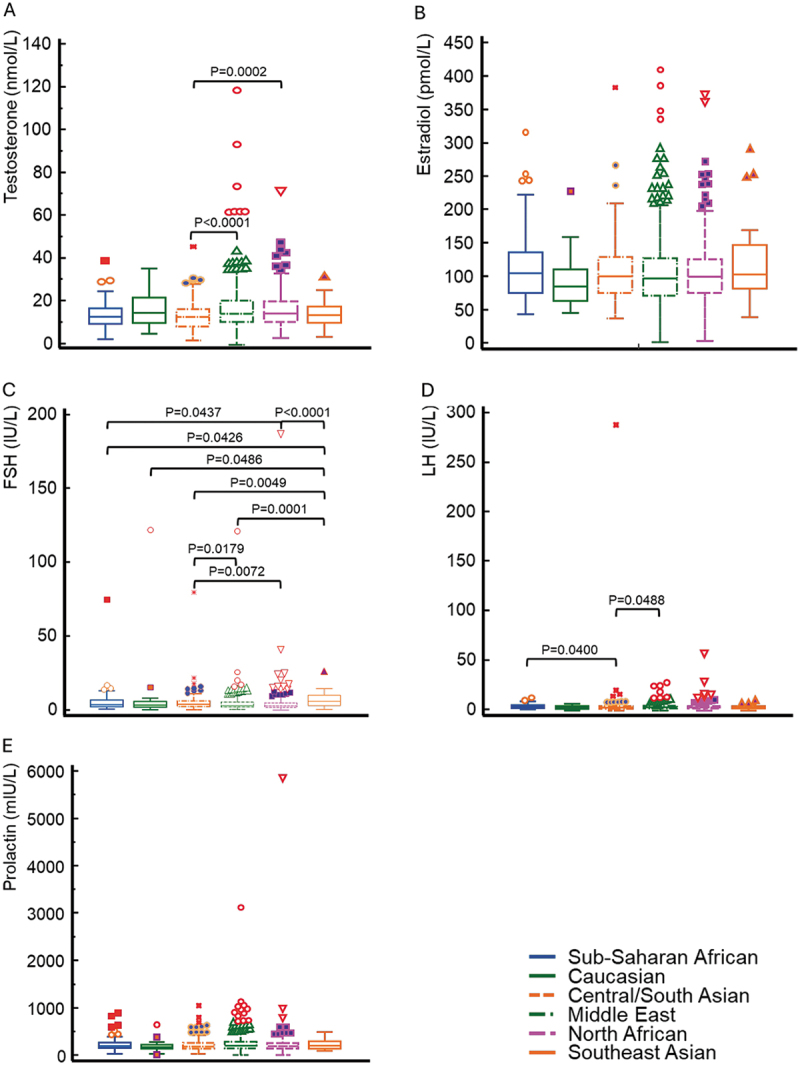
Box-and-whisker plots of testosterone (a), estradiol (b), follicle stimulating hormone (FSH) (c), luteinizing hormone (LH) (d), and prolactin (e) of men from different regions. Some significant differences between men from different regions are obvious of testosterone, FSH and LH, but not estradiol and prolactin.

The distribution for low/normal/high values of estradiol, FSH, LH, prolactin, and serum testosterone is also depicted in Table 2 and Figure 4. For the hormones investigated in this study, significant differences between different racial groups could only be observed for testosterone (between Central/South Asian and Northern African men) (Figure 4a), for FSH (Figure 4c) as well as for LH (between Sub-Saharan African, Central/South Asian and Middle Eastern men) (Figure 4d). For estradiol (Figure 4b) and prolactin (Figure 4e), no racial differences were observed in this study.

In order to investigate whether or not the observed differences are indeed due to racial background or due to age, logistic regression analyses were performed with patients’ age and racial descent (Sub-Saharan, Caucasian, Central/South Asian, North African, Southeast Asian) as independent variables. Results show that for the dependent variables, normozoospermia, oligozoospermia, asthenozoospermia, teratozoospermia, testosterone, FSH, sperm DNA fragmentation, and ORP, the overall fit of the model was significant, thus indicating that age and racial background contributed to the outcome. In this study, age contributed significantly to normo- (*p* = 0.0006), astheno- (*p* = 0.0007), and teratozoospermia (*p* = 0.0008). Racial descent was significant for normo- (Caucasian; *p* = 0.0204), oligo- (Central/South Asian; *p* < 0.0001), astheno- (Caucasian; *p* = 0.0274), and teratozoospermia (Caucasian, *p* = 0.0071; Central/South Asian, *p* = 0.0001).

For FSH, the model was also significant (*p* = 0.0276), but only for age (*p* = 0.0248) and Southeastern Asian men (*p* = 0.0069), whereas for testosterone a significant (*p* = 0.0033) contribution was found in Central/South Asian men. While sperm DNA fragmentation appears to be influenced by age (*p* < 0.0001), Central/South Asian (*p* = 0.0020) and Northern African (*p* = 0.0302) descent, no effect of age (*p* = 0.4909), Sub-Saharan African (*p* = 0.4178), and Southeastern Asian (*p* = 0.5232) descent could be observed for ORP, but for Caucasian (*p* = 0.0013), Central/South Asian (*p* < 0.0001), and Northern African descent (*p* = 0.0259). Although the overall model for sperm DNA fragmentation was significant with a comparably large Chi-squared value (15.2650), the Hosmer and Lemeshow test indicates a relatively poor fit of the logistic regression model for sperm DNA fragmentation (*p* = 0.0542). On the other hand, for normozoospermia, oligozoospermia, FSH, testosterone, and seminal ORP as oxidative stress parameter, the Hosmer and Lemeshow test indicates good fits of the logistic regression models with comparably smaller Chi-squared values (4.7083, 10.0814, 7.3107, 6.2356, and 9.0824, respectively) (*p* > 0.0542).

## Discussion

While categorizing people by race can be contentious due to potential discrimination and lack of genetic evidence [20], including a patient’s racial background in diagnostics may be clinically useful [21]. Personalized medicine benefits from considering racial influences on diagnostic parameters, as reproduction is a basic human right under Article 16:1 of the United Nations Human Rights Declaration [22]. Scholars argue that it is unwise to stop recording race when our understanding of the human genome is still developing [23].

In personalizing medicine, racial differences in endocrinology, urology, cardiology, and reproductive medicine should not be ignored. The American Heart Association’s Heart Failure Program shows that ‘black’ patients have a lower death risk post-hospital admission [24] and the National Center for Health Statistics reports higher heart disease deaths in Pacific Islander patients [25]. The Center for Disease Control and Prevention notes higher cardiovascular disease death rates in non-Hispanic black males compared to other groups [26]. Studies indicate that African Canadian men have higher FSH and testosterone levels, and black men have higher PSA levels and prostate cancer diagnoses [12,27]. Racial differences also affect ICSI program outcomes, with Mestizos having the highest fertilization and pregnancy rates and Asians the lowest [28].

In the present study, six different racial groups of males were investigated in one center in Doha, Qatar, a country where most of these men came to for economic/job purposes. This is where this study differs from most other studies as environmental effects that might influence the results are limited because of the relatively small size of the country. In addition, this study includes a wider variety of different races and investigates numerous parameters taken during andrological examinations including the hormones testosterone, estradiol, FSH, LH and prolactin, testicular volume, sperm concentration, motility, and normal sperm morphology. To the best of the knowledge of the authors, this is also the first study that includes functional parameters such as sperm DNA fragmentation and seminal oxidative stress as determined as oxidation–reduction potential (ORP) by means of the MiOXSYS analyzer. In contrast, other studies mainly focused on semen or on hormonal parameters.

Johnson et al. found lower parenchymal weight and smaller seminiferous tubules in Chinese men compared to Caucasian and Hispanic males, suggesting possible racial differences in total testicular volume [29]. However, these findings have low probability due to small sample sizes. Larger sample measurements of sperm concentration did not support these differences. Chang et al. reported lower total motile sperm count in African American men compared to White American men [30]. Redmon et al. found lower semen volume, sperm concentration, and total motile sperm count in Black Americans compared to White and Hispanic men [10]. Walker et al. noted similar lower values in infertile Black men, indicating a higher incidence of poor semen quality according to WHO criteria [31].

This study is the first to compare sperm DNA fragmentation and seminal oxidative stress in different racial groups. Caucasian men had the lowest mean percentage of sperm DNA fragmentation, while Central/South Asian men had the highest. Caucasian men also had the lowest probability of high fragmentation, with Central/South Asians having the highest. Due to small sample sizes, particularly in the groups of Caucasian and Southeast Asian men, however, these results should be viewed cautiously and need further verification. Similarly, caution needs to be taken for the testicular volume. Significant racial differences in seminal oxidative stress were observed, with Southeastern Asian and Sub-Saharan African men showing the highest levels. A larger study found no differences in SDF between MENA and non-MENA patients [13]. When categorizing the SDF data presented here into MENA and non-MENA patients, a similar result is obtained (*p* = 0.2144; not shown). Yet, for ORP the difference is obvious and significant (*p* = 0.0003; data not shown). Abate and Salini detected higher oxidative stress in blood samples in African soccer players as compared to Caucasians, a difference which was even more pronounced in February [32]. Since Black men are at higher risk of general oxidative stress and inflammation [33], our finding that Sub-Saharan African men have high seminal ORP values and the highest prevalence of seminal oxidative stress with high levels of SDF would be consequential. The reason for the higher oxidative stress levels in Sub-Saharan African men could be that Africans rather have a diet rich in carbohydrates which has been associated with oxidative stress [34,35]. On the other hand, genetic differences could also contribute to or cause different levels of oxidative stress and should not be neglected [36].

Reports on racial differences in male hormones are scarce. Most studies, primarily conducted in the USA, compare White, Black, Chinese, and Hispanic men [37,38]. Richard et al. reported higher testosterone levels in Black American men [37], while Litman et al. and Mazur found no differences [38,39]. In our study, testosterone levels in men Sub-Saharan African did not differ from those measured in all other racial groups. Contrary to Rajandram et al., we found no difference in serum testosterone between Southeast Asian and Central/South Asian men [40].

For estradiol, racial differences with significantly higher levels in Black men than in Caucasians have been reported before [37]. Similarly, Lopez et al. and Chadid et al. reported the highest median serum estradiol levels in Non-Hispanic Black men as compared to Non-Hispanic White and Mexican American men [41,42]. This finding was not confirmed in this study. However, if differences in the serum concentration of testosterone and estradiol are observed, they are reportedly associated with differences in lifestyle, diet, environmental, and/or genetic factors [43,44].

For the gonadotropins FSH and LH, we found significant racial differences with highest values in Southeast Asian, Central/South Asian, and Sub-Saharan African men, respectively. For prolactin, no differences between different groups were found. To the authors’ best knowledge, this is the first study comparing these hormones among men of different race groups with levels showing similar results as Manson and co-workers for FSH in women [45]. However, it must again be stressed that the sample sizes, specifically for Caucasian and Southeast Asian men were with 22 and 40, respectively, relatively small.

As a strength of this study, the inclusion of a variety of different racial groups has to be mentioned, not only a comparison of African, Hispanic, and White Americans. In addition, considering that only one center was recruiting the patients, it can be accepted that environmental influences on the parameters are limited as all men included in the study were exposed to the same environment in Qatar, a small country. Furthermore, this is the first study not only examining standard semen and sperm parameters, but also the functional parameters sperm DNA fragmentation and oxidative stress, as well as relevant hormones. On the other hand, this study also has its limitations such as the small groups of Caucasian and Southeast Asian men and the absence of East Asian (e.g. Chinese or Japanese) men has to be mentioned. The small sample size is particularly obvious for the testicular volume and sperm vitality as these parameters were only taken in 33, 26, and 7, and 57 of Sub-Saharan African, 20 Southeast Asian, and 5 Caucasian men, respectively. Such small sample sizes can result in unusual median values.

In conclusion, our results indicate significant racial differences in numerous male fertility parameters as supported by logistic regression and supported by the Hosmer/Lemeshow test. Hence, since standard semen analysis is not only limited due to biological variability, but there is also a racial factor that contributes to the outcome, a reference value that can be appropriate for one racial group might not be suitable for another group as this could have diagnostic and clinical consequences. Therefore, the concept of standardized globally valid reference values for all men should be revisited. Yet, additional and larger studies including functional sperm and semen parameters need to be conducted.

## Data Availability

Data not available due to ethical/legal restrictions.
